# Exploring the effect of different application rates of biochar on the accumulation of nutrients and growth of flue-cured tobacco (*Nicotiana tabacum*)

**DOI:** 10.3389/fpls.2024.1225031

**Published:** 2024-02-23

**Authors:** Yingfen Yang, Waqar Ahmed, Chenghu Ye, Linyuan Yang, Lianzhang Wu, Zhenlin Dai, Khalid Ali Khan, Xiaodong Hu, Xiaohong Zhu, Zhengxiong Zhao

**Affiliations:** ^1^ Yunnan Agricultural University, Kunming, Yunnan, China; ^2^ Yunnan Revert Medical and Biotechnology Co., Ltd., Kunming, Yunnan, China; ^3^ Nujiang Green Spice Industry Research Institute, Lushui, Yunnan, China; ^4^ Center of Bee Research and its Products/Unit of Bee Research and Honey Production/Research Center for Advanced Materials Science (RCAMS) and Applied College, King Khalid University, Abha, Saudi Arabia

**Keywords:** carbon/nitrogen ratio, nutrients and dry matter accumulation, plant growth stages, soil organic matter (SOM), tobacco

## Abstract

**Background:**

Biochar application has become one of the most potential tools to improve soil fertility and plant growth for sustainable and eco-friendly agriculture. However, both positive and negative effects of biochar application have been recorded on plant growth and soil fertility.

**Methods:**

This study investigated the impact of different application rates (0, 600, 900, 1200, and 1800 kg/ha) of biochar on the soil nutrient contents, accumulation of nutrients and dry matter in different plant parts, and growth of flue-cured tobacco plants under field conditions.

**Results:**

Results demonstrated that soil organic carbon pool and carbon/nitrogen ratio were increased proportionally with the increasing dosage of biochar, 25.54 g/kg and 14.07 g/kg compared with control 17 g/kg and 10.13 g/kg, respectively. The contents of soil total nitrogen were also significantly increased after biochar application in the middle (1.77 g/kg) and late-growth (1.54 g/kg) stages of flue-cured tobacco than in control (1.60 g/kg and 1.41 g/kg, respectively). The contents of soil nitrate nitrogen were also higher under low (600 and 900 kg/ha) application rates of biochar and reduced when higher (1200 and 1800 kg/ha) dosages of biochar were applied. However, it was observed that varying application rates of biochar had no impact on soil ammonium nitrogen content during the growth period of flue-cured tobacco plants. The nutrient accumulation (N, P, K) in different parts of flue-cured tobacco plants was significantly increased under a low application rate of biochar, which enhanced the soil and plant analyzer development values, effective leaves number, growth, dry matter accumulation, and leaf yield of flue-cured tobacco. In contrast, the high biochar application rate (1200 and 1800 kg/ha) negatively impacted nutrient accumulation and growth of flue-cured tobacco.

**Conclusion:**

Conclusively, the optimum application of biochar (600 and 900 kg/ha) is beneficial for plant growth, soil fertility, accumulation of nutrients, and dry matter in different plant parts. However, excessive biochar application (> 900 kg/ha) could inhibit flue-cured tobacco plant growth. This study provides a theoretical foundation for biochar application in tobacco and other crop production to obtain agricultural sustainability and economic stability.

## 1 Introduction

Biochar is a carbon-rich (65-90%) substance produced by the thermal decomposition of biomass (agricultural, forestry, and domestic waste as well as animal residues) at high temperatures (<700 °C) under complete or partial anaerobic conditions (Gabhane et al., 2020; Li et al., 2022). It is characterized by a large specific surface area, rich pore space, and high adsorption capacity (Tripathi et al., 2016). In organic farming, biochar is commonly used as a soil conditioner that regulates soil health, activity of soil microorganisms, nutrient uptake ability of plants, and yield (Schmidt et al., 2014; Li et al., 2022). Soil organic carbon (SOC) is the primary carbon source, constituting more than twice the total carbon in the atmosphere (Lal, 2015). However, due to irregular agricultural practices, increasing temperature, and unexpected climate change, the SOC pool is dropping gradually and causing lower crop yield (Qambrani et al., 2017). Thus, soil amendments with biochar can increase the SOC pool (Zhan et al., 2015), functional diversity of soil microorganisms (Li et al., 2022), and improve the nutrients and water retention ability of soil (Lorenz et al., 2007; Singh et al., 2019). Furthermore, it can enhance plant photosynthesis and growth (Rizwan et al., 2018), chlorophyll- and N-balance index (Qian et al., 2019), uptake and utilization ability of nutrients, particularly nitrogen (N), phosphorus (P), and potassium (K) (Li et al., 2019; Zheng et al., 2020), as well as increase crop biomass and yield (Uzoma et al., 2011; Yu et al., 2019).

The effect of biochar on soil physicochemical properties and plant growth varies depending on the nature and application rate of the biochar (Yang et al., 2023). Biochar amendment in the soil at a certain level can promote the growth and biomass of flue-cured tobacco, while higher application of biochar harms plant growth (Zhang et al., 2016). In addition, soil amendments with biochar significantly suppressed the incidence of soil-borne diseases (Alaylar et al., 2021) such as damping-off (Jaiswal et al., 2017), bacterial wilt (Gao et al., 2019), root rot (Jaiswal et al., 2019), and black shank (Zhang et al., 2021), by improving soil microbial diversity and soil health, induction of plant systemic resistance, and reducing the pathogen load in the plant rhizosphere (Iacomino et al., 2022). Previous studies have reported that a 1–4% application rate of biochar improves soil health and enhances plants’ N, P, and K uptake ability and yield (Singh et al., 2015). However, when the dose of biochar increases (>5%), it inhibits the growth of the plant and disturbs the soil’s physicochemical properties (Pokovai et al., 2020; Zheng et al., 2020; Li et al., 2022). Rajkovich et al. (2012) identified a threshold level of 2% (equivalent to 26 t/ha) biochar for maize crop and reported that >2% application of biochar results in stunted growth of maize. Similarly, Li et al. (2022) reported that a 2% application of biochar significantly mitigates the incidence of tobacco bacterial wilt disease by improving the functional diversity of rhizosphere microorganism and reducing the population of *Ralstonia solanacearum* in the rhizosphere of flue-cured tobacco plants.

In contrast, the high application rate of biochar can reduce soil mineral nutrient availability in the short term (Van Zwieten et al., 2010; Tammeorg et al., 2014) and disturb the soil microbial biomass and activity (Ameloot et al., 2014). These adverse effects may hinder seed germination and inhibit crop growth, drastically reducing crop yield (Chan et al., 2007). The excessive use of biochar reduces biomass accumulation and yield, but the underlying mechanism remains elusive. Flue-cured tobacco (*Nicotiana tabacum* L.) is an important industrial crop in Yunnan Province, China, and Yunnan produces about 50% of the total tobacco leaf yield in China (Ahmed et al., 2022a). Yunnan produces superior-quality tobacco that is famous for its rich taste, golden color, and fragrant aroma (Tang et al., 2020). However, the flue-cured tobacco yield and quality in Yunnan Province are affected due to continuous monocropping, inadequate supply of nutrients, and attacks of diseases and insect pests (Ahmed et al., 2022b). Therefore, developing sustainable agricultural approaches in the monocropping regions are essential for better yield and high-quality production of flue-cured tobacco leaves. Our previous study demonstrated that biochar application at a certain level (2%) significantly suppressed the incidence of tobacco bacterial wilt disease (Li et al., 2022). However, the impact of different application rates of biochar on soil nutrients, tobacco plant growth, biomass accumulation, and yield in the monocropping areas is still unknown.

We hypothesized that a higher dosage of biochar may disturb the balance among soil physicochemical properties, nutrient availability, and assimilation by plants, thereby inhibiting growth. Therefore, the present study explored the impact of different application rates (0, 600, 900, 1200, and 1800 kg/ha) of biochar on soil nutrient contents, accumulation of nutrients and dry matter in different plant parts, growth of flue-cured tobacco plants at different growth stages. The main objective of this study is to quantify the adverse effects of high application rates of biochar on the growth and nutrient accumulation of flue-cured tobacco plants. This study provides an experimental and theoretical knowledge on the efficient use of biochar for the better production of flue-cured tobacco.

## 2 Materials and methods

### 2.1 Experimental site

Two field experiments were conducted during the growing season from April to September 2021 at Mouding County, Chuxiong City (25° 1′ 58.8″ N, 101° 32′ 45.24″ E) and Malong County, Qujing City (25° 29′ 27.6″ N, 103° 47′ 45.6″ E), Yunnan Province, China. The average annual temperature of 15.8 °C, annual rainfall of 872 mm, an average of 238 frost-free days, and 2359 hours of sunshine per year were recorded at the Chuxiong experimental site. At the Qujing experimental site, the average annual temperature, annual rainfall, average frost-free days, and sunshine hours per year were recorded as 14.3 °C, 927.1 mm, 234 days, and 2158 hours, respectively. The basic physicochemical properties of soil at both experimental sites are listed in **Table 1**.

**Table 1:** Basic physicochemical properties of soil at both experimental sites.

Experimental site	Soil type	SOC (g/kg)	TN (g/kg)	AN (mg/kg)	AP (mg/kg)	AK (mg/kg)
Chuxiong	paddy soil	21.21	2.34	137.9	7.81	182.5
Qujing	red earth	12.81	1.14	92.00	22.85	180.7

Here: SOC, Soil organic carbon; TN, total nitrogen; AN, available nitrogen; AP, available phosphorus, and AK, available potassium.

### 2.2 Preparation of biochar

Biochar used in this study was prepared using flue-cured tobacco stem at slow pyrolysis (= 450 °C) with 4 h residence time using a muffle furnace by Kunming Canghui Co. Ltd. Yunnan, China. The resulting biochar was sieved through a 3 mm mesh prior to use. The basic physicochemical properties of biochar were as follows: pH (10.16), total carbon (57.83%), total nitrogen (2.05%), total phosphorus (1.24%), total potassium (3.65%), total cation exchange (12.07 cmol/kg), electrical conductivity (3.68 ms/cm), bulk density (0.23 g/cm^3^), and ash contents (28.9%).

### 2.3 Field trials and design description

**Figure 1:**
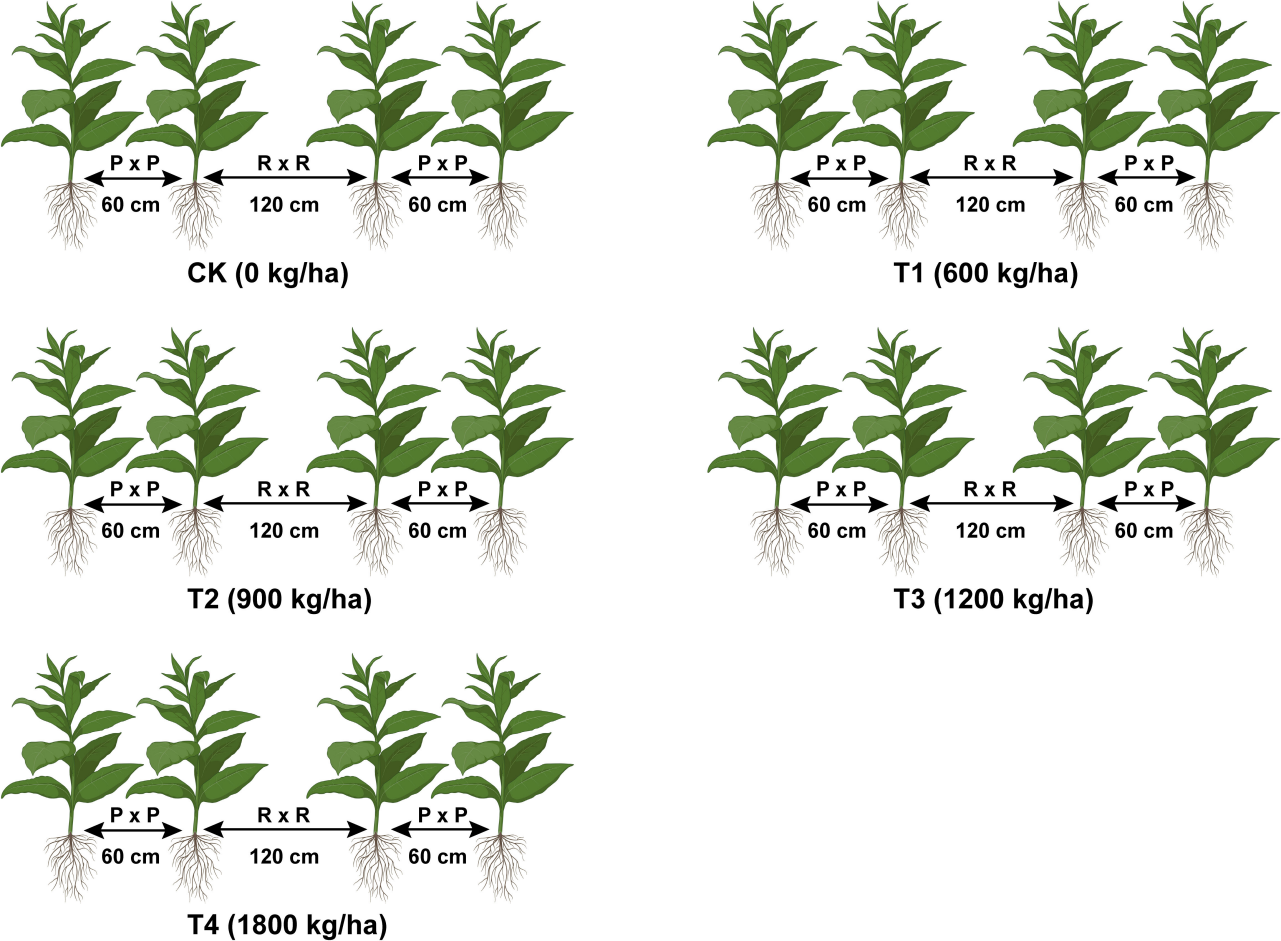
Schematic diagram of experimental conditions under application rates of biochar. Biochar was mixed thoroughly in the holes on ridges before seedling transplantation. Biochar application rates: 0 kg/ha (CK), 600 kg/ha (T1), 900 kg/ha (T2), 1200 kg/ha (T3), and 1800 kg/ha (T4).

Field trials were conducted using the flue-cured tobacco cultivars Yun87 and Yun121 from April to September 2021 in Chuxiong and Qujing cities, Yunnan, China. In April 2021, the field was prepared for tobacco cultivation by raising ridges and applying biochar and fertilizer. Biochar and fertilizer (base fertilizer) were thoroughly mixed with the soil in holes before seedling transplantation. In the first week of May 2021, seedlings (50 days old) of flue-cured tobacco cultivars Yun87 and Yun121 were transplanted on ridges (in rings) having a plant-to-row spacing of 55 × 120 cm at Chuxiong and Qujing experimental sites, respectively and experiment was performed under five conditions (**Figure 1**). To overcome the nutrient deficiency, fertilizers were applied as base and top (70:30); 70% of the total base fertilizer was applied before transplantation of seedlings, whereas 30% of top fertilizer was used three times within 35 days after seedling transplantation (Cai et al., 2021). At the Chuxiong experimental site, 105 kg/ha of pure nitrogen and 600 kg/ha of tobacco-specific fertilizer (N-P_2_O_5_-K_2_O; 15-15-18) were applied as N: P: K (1:2:2.5). In contrast, at Qujing experimental site, 105 kg/ha of pure nitrogen and 600 kg/ha of tobacco-specific fertilizer (N-P_2_O_5_-K_2_O; 15-8-25) were applied as N: P: K (1:1:3). The integrated field management methods were adopted at both experimental sites according to the National Standards of Tobacco Industry in China (Song et al., 2022). The experiment was carried out under a randomized complete block design and repeated thrice with 15 plots (Plot size = 6 m × 7.4 m per replication; 3 plots per treatment), and each plot contained 65 tobacco plants.

### 2.4 Soil samples collection and analysis of soil physicochemical properties

The soil samples were collected in replicates from each treatment at different growth stages of flue-cured tobacco plants after 35 (late reunion stage), 60 (late vigorous growth stage), and 85 (early baking stage) days of transplantation from both experimental sites. Soil samples were collected at a depth of 10-30 cm around the stem of flue-cured tobacco plants within a diameter of 15 cm using a shovel (5 cm in diameter) following the S-type sampling methods from each treatment. Briefly, 10 cores of soil samples were collected from each plot per treatment and mixed thoroughly to make one sample, and a total of three samples were collected from each treatment. The soil samples were sieved through a 2-mm mesh, placed in sterilized bags, and delivered to the laboratory for further study. The contents of soil organic carbon (SOC; g/kg) and total nitrogen (TN; g/kg) were determined by using the K_2_Cr_2_O_7_ oxidation external heating method and elemental analyzer (Elementar Analysensysteme GmbH, Germany), respectively (Wang et al., 2022). Soil nitrate-nitrogen (NO_3_^−^−N; mg/kg) and ammonium nitrogen (NH_4_^+^−N; mg/kg) were extracted with indophenol-blue colorimetric and 1 M KCl methods, and their concentrations were measured at OD_275_ and OD_220_ nm using a spectrophotometer (UV-6000, China), respectively (Zhao et al., 2017). The soil carbon/nitrogen (C/N) ratio was calculated using the following formula:


C/N=Soil organic carbon(SOC)/total nitrogen(TN)

### 2.5 Determination of agronomic traits

The agronomic traits of flue-cured tobacco plants including plant height (cm), stem circumference (cm), number of effective leaves, maximum leaf length (cm), maximum leaf width (cm), and maximum leaf area (cm^2^) from each treatment after 35, 60, and 85 days of transplantation, were recorded according to the “Tobacco Industry Standard YC/T 142-1988 Tobacco Agronomic Trait Survey Methods” in China (Pan et al., 2015). Briefly, data were collected from 10 plants from each plot per treatment, and the mean value was calculated. The leaf area (cm^2^) was calculated using the following formula (Suggs et al., 1960): leaf length × leaf width × 0.6345.

### 2.6 Analysis of soil and plant analyzer development value of tobacco leaves

The soil and plant analyzer development (SPAD) value of flue-cured tobacco plants were calculated from the mid to the late maturing stage after 45, 60, 75, and 90 days of transplantation from each treatment. Briefly, 10 plants were randomly selected from each plot per treatment, and the 5^th^ leaf per plant was selected from top to bottom after 45 days of transplantation to calculate the SPAD value by using the Spad-520 PLUS portable chlorophyll meter (Konica Minolta, Japan) (Gao et al., 2017). The 10^th^ leaf from top to bottom was selected after 60, 75, and 90 days of transplantation to calculate the SPAD value. Each tobacco leaf was measured at 6 points on the symmetry on both sides, 3 cm from the central vein, and the average value was recorded.

### 2.7 Assessment of the accumulation of dry matter, total nitrogen, total phosphorus, and total potassium contents of tobacco plants

The accumulation of dry matter contents (g/plant) in different parts (root, stem, and leaf) of flue-cured tobacco plants was determined at 35, 60, and 85 days of post-transplantation under different treatments. Briefly, 5 plants were randomly uprooted per plot from each treatment and divided into three parts (root, stem, and leaf). The collected samples were incubated at 105 °C for 30 min, dried to constant weight at 80 °C for 48 h, and the contents of dry matter were measured in each part (Li et al., 2022). The contents (mg/plant) of nitrogen (N), phosphorus (P), and potassium (K) were determined in different parts (root, stem, and leaf) of flue-cured tobacco plants from the samples collected after 85 days of transplantation. The samples were digested with H_2_SO_4_-H_2_O_2,_ and the contents of N, P, and K were determined with continuous flow analyzer, molybdenum-antimony anti-colorimetric, and flame photometric methods, respectively (Zhai et al., 2013). The whole plant dry matter accumulation (g/plant), dry matter accumulation ratio in each part (%), whole plant nutrients accumulation (mg/plant), nutrients accumulation in each part (mg/plant), and nutrients accumulation ratio in each part (%) were calculated using the following formulae:


$$\begin{array}{c} \text{Whole plant dry matter accumulation}\left(\text{g/plant}\right)\\ =\Sigma \;(\text{dry matter accumulation of root, stem, and leaf}) \end{array}$$

$$\begin{array}{c} \text{Dry matter accumulation ratio in the root, stem, and leaf}\left(\%\right)\\ =\left(\text{dry matter contents of each organ/whole plant dry matter accumulation}\right)\times 100 \end{array}$$

$$\begin{array}{c} \text{Whole plant nutrients accumulation}\left(\text{mg/plant}\right)\\ =\Sigma \;(\text{nutrients accumulation of root, stem, and leaf}) \end{array}$$

$$\begin{array}{c} \text{Nutrient accumulation in each part}\left(\text{mg/plant}\right)\\ =(\text{dry matter contents of an organ of the tobacco plant at }85\text{ days of post-transplantation}\\ \times \text{ contents of N, P, and K in an organ of the tobacco plant at }\\ 85\text{ days of post-transplantation}) \end{array}$$

$$\begin{array}{c} \text{Accumulation ratio of N, P, and K in the root, stem, and leaf}\left(\%\right)\\ =(\text{contents of N, P, and K in an organ of the tobacco plant at }\\ 85\text{ days of post-transplantation}/\text{contents of N, P, and K in the whole plant at }\\ 85\text{ days of post-transplantation})\times 100 \end{array}$$

### 2.8 Statistical analysis

Microsoft Excel 2019 software was used to sort out data, and they were statistically analyzed using analysis of variance (ANOVA) in SPSS version 22.0 software (IBM, Chicago, USA). The significant difference among treatments was calculated by the least significant difference (LSD) and was considered significant when *p<* 0.05. To further investigate the impact of biochar dosage on tobacco plant growth and nutrient accumulation, a correlation analysis was performed between biochar dosage, agronomic traits (plant height, stem circumference, number of effective blades, and leaf length, width, and area), nutrients accumulation (N-P-K) in whole plant and plant parts (leaf, stem, and root), and soil physicochemical properties (SOC, TN, C/N ratio, NO_3_^−^−N, and NH_4_^+^−N) according to Spearman correlation (*p*< 0.05) using the ggcor package in “ggplot2” and results were visualized through a heatmap. All figures were visualized in GraphPad_Prism (8.0.2) and were adjusted and combined using Adobe Illustrator 2019.

## 3 Results

### 3.1 Biochar regulates the growth of flue-cured tobacco plants

Results demonstrated that plant height, effective leaf number, and maximum leaf area were significantly higher in CK (0 kg/ha) compared with T1-T4 (T1:600 kg/ha, T2:900 kg/ha, T3:1200 kg/ha, and T4:1800 kg/ha) after 35 days of transplantation at both experimental sites (**Table 2**). At 60 days of post-transplantation, the plant height and the number of effective leaves under treatment T3 and T4 were all lower than CK, but all other indices were higher than those of CK. However, after 85 days of post-transplantation, the agronomic trait indices under T1 and T2 were significantly higher than CK, T3, and T4 (LSD; *p <* 0.05). This indicates that the application of biochar at a specific dosage is beneficial to the growth of flue-cured tobacco plants. In contrast, excessive biochar application inhibits the growth of flue-cured tobacco plants.

**Table 2:** Effect of different application rates of biochar on the agronomic traits of the flue-cured tobacco plants.

Experimental site	Days after transplantation	Treatments	Plant height (cm)	Stem circumference (cm)	Number of effective leaves	Maximum leaf length (cm)	Maximum leaf width (cm)	Maximum leaf area (cm^2^)
Chuxiong	35	CK	18.33±0.29a	5.63±0.81a	11.67±0.58a	35.57±2.23a	17.50±1.76a	396.57±63.99a
		T1	14.60±0.66b	4.60±0.60a	11.00±1.00ab	30.07±0.93ab	15.70±0.82a	299.61±20.71ab
		T2	14.33±0.58b	5.17±0.21a	11.33±0.58ab	33.83±1.70bc	16.53±2.21a	356.12±60.84ab
		T3	14.00±0.50b	5.00±0.87a	11.33±0.58ab	31.40±1.82bc	15.17±0.61a	302.55±28.49ab
		T4	12.27±1.17c	4.53±0.31a	10.00±1.00b	30.70±2.79c	14.60±2.54a	286.80±72.63b
	60	CK	93.00±1.00ab	7.33±0.28a	19.00±1.00ab	60.33±2.52c	29.00±1.00ab	1109.74±46.90bc
		T1	96.33±2.52a	7.77±0.68a	20.00±2.65a	69.33±2.08a	32.33±4.04a	1421.07±169.51a
		T2	94.00±2.00ab	7.70±0.82a	19.68±2.08ab	66.33±2.52ab	28.67±5.51ab	T3.69±187.24ab
		T3	89.00±2.65b	7.90±0.36a	18.67±2.52ab	61.33±4.73bc	30.67±2.89ab	1197.30±188.81ab
		T4	79.33±4.93c	7.70±0.26a	16.33±1.15b	54.33±1.15d	24.67±1.15b	850.65±51.40c
	85	CK	110.00±3.00bc	9.10±0.37a	21±0.84ab	71.67±1.53b	28.67±1.53b	1303.05±61.35bc
		T1	119.33±4.16a	9.50±0.50a	21.87±0.75a	75.50±0.5a	34.63±2.47a	1659.42±124.88a
		T2	114.83±2.84ab	9.37±1.01a	21.4±0.69a	73.83±1.04ab	32.73±3.16a	1534.82±169.56ab
		T3	107.33±2.52cd	8.83±0.29a	19.73±0.64bc	66.93±1.91c	27.53±2.36b	1167.58±71.11cd
		T4	103.33±1.53d	8.5±0.50a	18.57±0.74c	64.50±1.8d	25.00±1.00b	1023.34±56.69d
Qujing	35	CK	17.97±0.65a	6.93±0.42a	13.78±1.34a	41.43±0.49a	17.66±1.89a	464.20±50.01a
		T1	17.80±1.79a	7.67±0.79a	13.55±0.84a	41.76±0.32a	17.50±1.05a	463.59±28.79a
		T2	17.70±0.36a	7.39±0.30a	13.22±1.02a	41.21±2.17a	17.18±1.23a	449.84±49.95a
		T2	17.13±0.97a	6.75±0.42a	12.44±0.51a	41.56±0.96a	18.20±1.37a	479.89±37.34a
		T3	15.73±0.21b	6.74±0.89a	11.56±0.77a	40.19±1.04a	16.86±1.47a	429.31±26.56a
	60	CK	106.67±2.89b	11.63±0.78ab	22.67±2.31a	82.67±1.53b	32.67±3.21ab	1713.36±171.65ab
		T1	119.33±3.51a	12.83±0.29a	23.33±1.53a	86.67±1.53a	35.00±3.00a	1923.38±144.00a
		T2	111.67±5.69ab	11.17±0.76b	23.00±4.36a	83.73±1.10ab	32.65±1.53ab	1734.85±59.65ab
		T3	98.33±2.52c	11.97±0.95ab	22.33±4.00a	83.00±2.65b	29.34±2.52b	1542.05±84.13b
		T4	96.33±5.86c	11.37±0.55b	22.00±5.20a	82.00±3.00b	29.33±3.79b	1525.55±197.6b
	85	CK	125.33±8.74b	12.93±1.01a	23.00±1.00a	85.20±2.03a	33.83±1.61a	1828.99±97.58a
		T1	142.00±6.00a	13.47±1.00a	24.00±1.00a	90.67±3.62a	36.17±3.55a	2085.02±274.58a
		T2	134.00±2.00a	12.80±0.53a	23.67±1.53a	90.17±4.01a	34.70±2.31a	1989.09±219.77a
		T3	119.00±5.00c	13.37±1.00a	23.33±1.15a	86.00±4.77a	33.60±2.01a	1836.89±199.93a
		T4	111.17±8.13c	12.53±0.85a	22.50±0.50a	84.43±2.00a	32.20±2.55a	1726.24±161.11a

Here: CK, T1, T2, T3, and T4 represent 0, 600, 900, 1200, and 1800 kg/ha application of biochar, respectively. The significant difference among treatments after specific days of post-transplantation is shown by different lowercase letters within a column according to the least significant difference test (LSD; p< 0.05).

### 3.2 Soil and plant analyzer development values of flue-cured tobacco leaves

**Figure 2:**
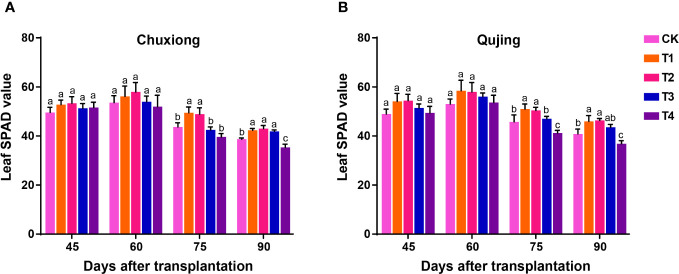
Effect of different application rates of biochar on soil and plant analyzer development (SPAD) value of flue-cured tobacco plants. Leaf SPAD values of flue-cured tobacco plants at Chuxiong **(A)** and Qujing **(B)** experimental sites. After specific days of transplantation, the significant difference among treatments (As described in **Figure 1**) is shown by different small letters on the error bars according to the least significant difference test (LSD; *p*< 0.05).

The soil and plant analyzer development (SPAD) values of flue-cured tobacco leaves were calculated after 45, 60, 75, and 90 days of transplantation at both experimental sites (**Figure 2**). The SPAD values of flue-cured tobacco leaves were recorded maximum at 60 days post-transplantation at both experimental sites and decreased with time. No significant difference was observed among the treatments in the SPAD value of flue-cured tobacco leaves after 45 and 60 days of post-transplantation at both experimental sites (LSD; *p* > 0.05). The SPAD value of flue-cured tobacco leaves in T1 (600 kg/ha) and T2 (900 kg/ha) were significantly higher than CK (0 kg/ha), T3 (1200 kg/ha), and T4 (1800 kg/ha) at 75 days of post-transplantation. At both experimental sites, no significant difference was observed in the SPAD value of flue-cured tobacco leaves after 90 days of transplantation in T1, T2, and T3, but the values were significantly higher than CK and T4 (**Figures 2A, B**). These results demonstrate that the optimum application of biochar could delay the degradation rate of chlorophyll and, therefore, prolong the maturation period of tobacco leaves, which is beneficial for the accumulation of dry matter in flue-cured tobacco leaves.

### 3.3 Application of biochar improves the accumulation and distribution of dry matter in flue-cured tobacco plants

At both experimental sites, the accumulation of dry matter in the whole plant and different parts was higher in CK (0 kg/ha) than T1-T4 (T1:600 kg/ha, T2:900 kg/ha, T3:1200 kg/ha, and T4:1800 kg/ha), at 35 days of post-transplantation (**Table 3**, LSD; *p<* 0.05), and no significant difference was observed among treatments (T1-T4) at Qujing experimental site. In addition, the distribution ratio of dry matter was in the order of leaf > root > stem, excluding T3 and T4 at Qujing experimental site. At 60 days post-transplantation, the dry matter accumulation in roots, stems, leaves, and whole plants under T1 was significantly higher than CK and other treatments (T2-T4) at both experimental sites. The dry matter accumulation in leaves and whole plants under T2 was higher than CK, and the dry matter distribution ratio was in the order of leaf > stem > root. At 85 days post-transplantation, the dry matter accumulation in the tobacco plants’ leaves, roots, and whole plants under T1 was significantly higher than CK and other treatments (T2-T4) at both experimental sites. However, the leaf dry matter accumulation of the tobacco plants in T2 was higher than that of CK, and the distribution ratio of dry matter was in the order of leaf > stem > root under all treatments at both experimental sites.

**Table 3:** Effect of different application rates of biochar on accumulation and distribution of dry matter in the flue-cured tobacco plants.

Experimental site	Days after transplantation	Treatments	Dry matter accumulation (g/plant)	Dry matter accumulation (g/plant)			Dry matter distribution ratio (%)		
				Root	Stem	Leaf	Root	Stem	Leaf
Chuxiong	35	CK	20.60±0.20a	2.62±0.15a	3.63±0.11a	14.34±0.23a	12.74±0.84a	17.64±0.37b	71.64±0.47a
		T1	19.67±0.03b	2.16±0.08b	3.18±0.07b	14.33±0.08a	10.98±0.39b	16.18±0.37c	71.51±0.76a
		T2	19.28±0.79bc	2.13±0.14b	3.26±0.08b	13.90±0.78a	11.06±0.84b	16.92±0.65b	71.58±0.11a
		T3	18.87±0.19c	2.21±0.04b	3.26±0.08b	13.39±0.25a	11.72±0.29ab	17.30±0.53b	71.62±0.23a
		T4	14.60±0.09d	1.44±0.07c	2.83±0.04c	10.34±0.15b	9.84±0.46c	19.36±0.38a	70.80±0.73a
	60	CK	134.23±1.70b	21.67±1.62b	26.83±3.37b	85.72±1.55b	16.14±1.16ab	19.98±2.34b	63.88±1.89a
		T1	173.24±4.70a	31.11±0.52a	43.53±3.30a	98.59±3.63a	17.97±0.53a	25.12±1.50a	56.91±1.68b
		T2	138.15±1.69b	19.52±0.35bc	30.18±2.84b	88.45±1.69ab	14.13±0.13b	21.84±1.83ab	64.04±1.81a
		T3	115.97±6.83c	17.63±1.42cd	25.35±3.11b	72.99±4.90c	15.20±0.61b	21.87±2.56ab	62.94±1.96a
		T4	112.63±14.53c	17.00±1.61d	24.22±4.69b	71.42±11.47c	15.24±2.14b	21.45±2.55ab	63.31±3.91a
	85	CK	255.60±30.82c	35.25±6.41c	52.83±5.15b	167.52±25.1b	13.72±0.92a	20.90±3.56a	65.38±2.74a
		T1	354.86±45.08a	62.81±4.93a	75.38±8.82a	216.67±25.69a	17.61±3.35a	21.27±1.12a	61.11±2.23a
		T2	318.99±20.98b	44.47±1.96bc	69.28±6.55a	205.24±13.49a	13.96±0.63a	22.00±2.15a	64.04±1.69a
		T3	245.46±10.46cd	48.75±3.45b	55.15±1.61b	141.55±9.32c	19.35±7.15a	22.63±2.09a	58.02±5.24b
		T4	218.82±7.16d	34.60±3.37c	51.01±2.97b	133.21±6.14c	15.32±6.84a	23.42±1.78a	61.26±5.76a
Qujing	35	CK	35.87±2.04a	5.30±0.93a	5.11±1.02ab	25.46±0.12a	14.71±1.75a	14.16±2.07bc	71.13±3.74a
		T1	35.17±1.17a	4.86±0.51a	5.45±0.07ab	24.86±0.62a	13.80±0.99a	15.50±0.39ab	70.71±0.61a
		T2	36.38±1.58a	4.86±0.88a	6.18±0.58a	25.34±0.49a	13.30±1.83a	16.98±1.01a	69.73±2.69a
		T3	35.19±0.47a	5.29±0.26a	4.57±0.12b	25.34±0.61a	15.03±0.81a	12.99±0.43c	71.99±0.81a
		T4	35.24±2.00a	5.41±0.94a	4.89±0.60b	24.94±0.81a	15.30±1.88a	13.83±0.94bc	70.87±2.80a
	60	CK	225.76±12.90c	33.92±4.50a	54.15±4.55a	137.69±4.55c	14.98±1.17a	23.96±0.67ab	61.05±1.84ab
		T1	260.03±1.14a	33.45±4.71a	61.95±4.33a	164.62±4.30a	12.87±1.86b	23.82±1.63ab	63.31±1.41a
		T2	243.54±6.46b	27.97±3.94a	61.43±2.76a	154.14±3.37b	11.46±1.33c	25.23±1.32a	63.30±1.10a
		T3	220.51±10.84c	34.23±1.40a	49.31±6.63a	136.97±6.15c	15.55±1.05a	22.32±2.17b	62.13±1.20ab
		T4	225.05±2.58c	33.05±4.29a	56.46±3.08a	135.54±3.78c	14.70±2.06a	25.08±1.08a	60.22±1.13b
	85	CK	518.09±21.37b	51.20±8.11a	137.52±8.71a	329.37±7.07b	9.66±1.03a	26.04±0.62b	64.30±1.45a
		T1	552.02±6.39a	55.73±4.79a	137.09±2.62a	359.20±6.72a	10.42±1.01a	24.34±0.85b	65.24±1.31a
		T2	517.57±14.15b	45.66±2.77a	134.97±17.65a	336.94±9.35b	8.95±1.30a	26.22±2.33b	64.83±2.36a
		T3	508.63±5.30bc	52.90±7.77a	148.50±1.04a	307.23±6.14c	10.25±1.34a	30.11±0.61a	59.64±1.86b
		T4	489.09±8.68c	52.65±1.66a	136.84±7.41a	299.60±5.57c	10.22±0.63a	31.67±1.41a	58.11±0.84b

Here: CK, T1, T2, T3, and T4 represent 0, 600, 900, 1200, and 1800 kg/ha application of biochar, respectively. Different lowercase letters within a column represent the significant differences among treatments according to the least significant difference test (LSD; p< 0.05) after specific days of transplantation.

### 3.4 Accumulation and distribution of nitrogen in flue-cured tobacco plants under different dosages of biochar

**Figure 3:**
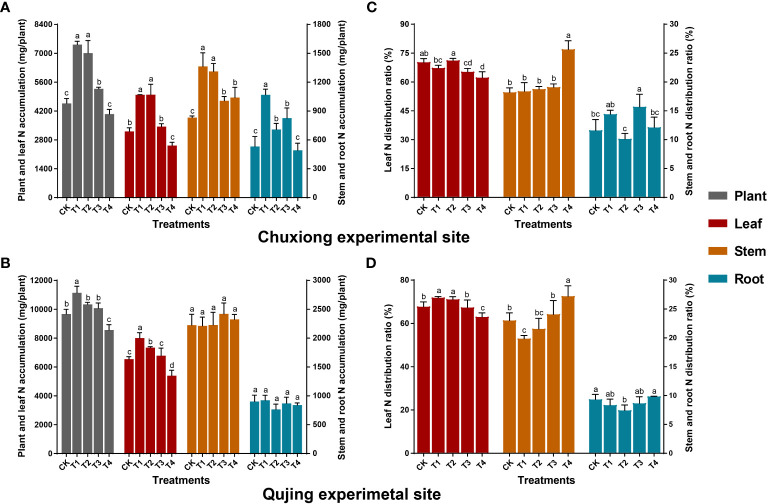
Impact of different biochar dosages on nitrogen (N) accumulation and distribution ratio in flue-cured tobacco plants. The top **(A-C)** and bottom **(B-D)** bar plots demonstrate the N accumulation and distribution ratio in *N. tabacum* at the Chuxiong and Qujing experimental sites, respectively. On the error bars, different small letters after specific days of transplantation show the significant difference among treatments (As described in **Figure 1**) according to the least significant difference test (LSD; *p*< 0.05).

The N accumulation in leaves and the whole plant first increased and then decreased with an increase in biochar application rate at both experimental sites (**Figures 3A, B**). At Chuxiong, the N accumulation in leaves, stems, and the whole plant in T1 (600 kg/ha) and T2 (900 kg/ha) were significantly higher (LSD; *p<* 0.05) than CK (0 kg/ha), T3 (1200 kg/ha), and T4 (1800 kg/ha) (**Figure 3A**). However, the N accumulation in roots was significantly higher in T1 than in CK, T2, T3, and T4. While at the Qujing experimental site, the N accumulation in leaves and the whole plant was significantly higher in T1 than in CK and T2-T4 (**Figure 3B**), whereas there was no significant difference among treatments for root and stem. The N accumulation in leaves increased by up to 55.33 and 55.45% in Chuxiong and 22.34 and 12.21% in Qujing experiment sites under T1 and T2 compared with CK. Further N distribution analysis revealed that N was mainly distributed in the leaves of flue-cured tobacco plants at both experimental sites (**Figures 3C, D**).

### 3.5 Application of biochar influences the accumulation and distribution of phosphorus in flue-cured tobacco plants

**Figure 4:**
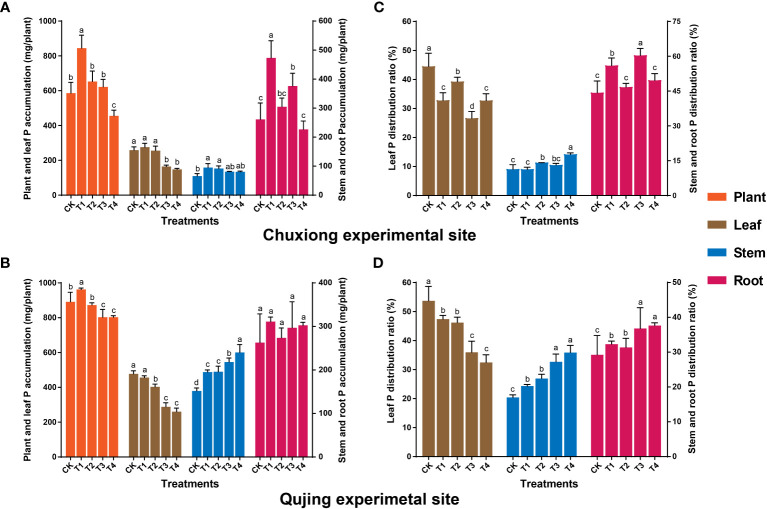
Effect of different biochar dosages on phosphorus (P) accumulation and distribution ratio in flue-cured tobacco plants. The top **(A-C)** and bottom **(B-D)** bar plots demonstrate the P accumulation and distribution ratio in flue-cured tobacco plants at the Chuxiong and Qujing experimental sites, respectively. The significant differences among treatments (As described in **Figure 1**) are shown by different lowercase letters on the error bars according to the least significant difference test (LSD; *p*< 0.05) after specific days of transplantation.

Results showed that P accumulation in the whole plant showed an increasing and decreasing trend with the increase of biochar application rate at both experimental sites. The P accumulation in the whole plant at Chuxiong and Qujing experimental sites under T1 increased by up to 44.06% and 7.94%, respectively, than CK (**Figures 4A, B**). At Chuxiong, the P accumulation was significantly higher in leaves of flue-cured tobacco plants treated with CK, T1, and T2 than in T3 and T4 (**Figure 4A**). However, the P accumulation in flue-cured tobacco leaves at the Qujing experimental site was significantly lower in T2, T3, and T4 compared with CK and T1 (**Figure 4B**). The P accumulation in the roots of flue-cured tobacco plants at the Chuxiong experimental site was significantly higher in T1 than CK and T2-T4. In contrast, no significant difference was observed among the treatments for P accumulation in the stem (**Figure 4A**). However, the P accumulation in the stem of flue-cured tobacco plants at the Qujing experimental site increased with the increase in the application rate of biochar and was significantly higher in T4 compared with CK and T1-T3, while no significant difference was observed among treatments for the roots (**Figure 4B**). Further distribution analysis showed that P was mainly distributed in the leaves and roots of flue-cured tobacco plants at both experimental sites (**Figures 4C, D**).

### 3.6 Biochar application affects the accumulation and distribution of potassium in flue-cured tobacco plants

**Figure 5:**
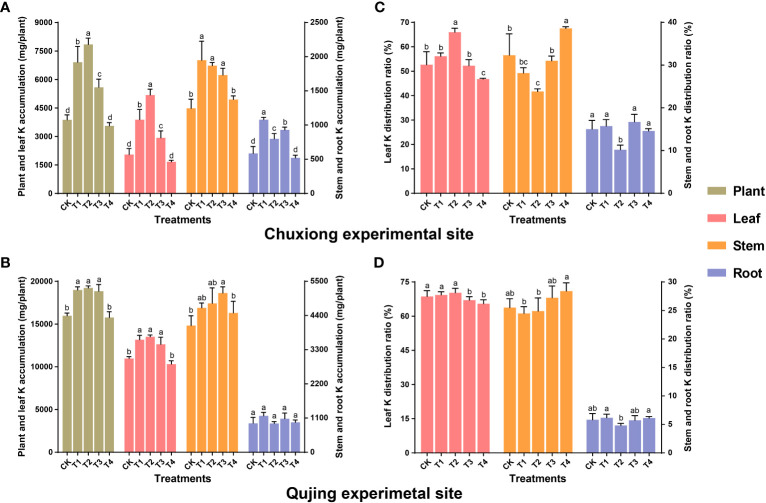
Impact of different biochar dosages on potassium (K) accumulation and distribution ratio in flue-cured tobacco plants. The top **(A-C)** and bottom **(B-D)** bar plots demonstrate the K accumulation and distribution ratio *N. tabacum* at the Chuxiong and Qujing experimental sites, respectively. The lowercase letters on the error bars represent the significant differences among treatments (As described in **Figure 1**) according to the least significant difference test (LSD; *p*< 0.05) after specific days of transplantation.

Results revealed that at both experimental sites, the K accumulation in leaves and whole plants increased first and then decreased with an increase in biochar application rate (**Figures 5A, B**). The K accumulation at Chuxiong experimental site in the leaves and whole plants under T2 was significantly higher than CK, T1, T3, and T4 (LSD; *p<* 0.05, **Figure 5A**). In contrast, at the Qujing experimental site, the K accumulation in the leaves and whole plants under T1, T2, and T3 was significantly higher than CK and T4 (**Figure 5B**). The K accumulation in leaves at the Chuxiong and Qujing experimental sites increased by up to 89.8, 153.03, 43.08% and 20.16, 23.27, 15.26% under T1, T2, and T3, respectively, as compared with CK. The K accumulation in the stem and root under T1 was significantly higher than CK at the Chuxiong experimental site (**Figure 5A**), whereas there was no significant difference among treatments for stem and root at the Qujing experimental site (**Figure 5B**). Further analysis shows that K is mainly distributed in the leaves and stems of flue-cured tobacco plants at both experimental sites (**Figures 5C, D**).

### 3.7 Impact of biochar application rates on the contents of soil organic carbon, total nitrogen, and carbon/nitrogen ratio

**Figure 6:**
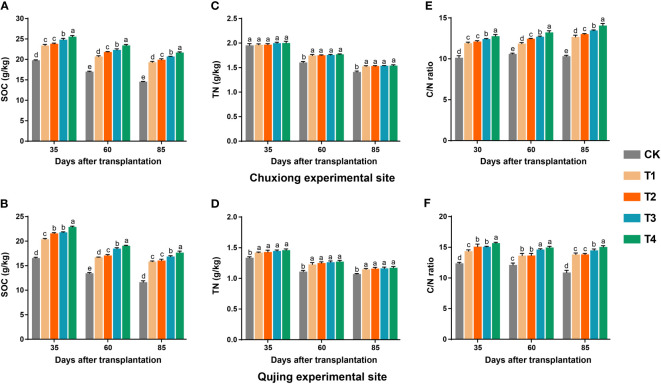
Effect of different biochar dosages on contents of soil organic carbon (SOC), total nitrogen (TN), and carbon/nitrogen ratio of flue-cured tobacco plants grown in paddy soil (Chuxiong) and red earth (Qujing). Bar plots top and bottom show the SOC **(A, B)**, TN **(C, D)**, and C/N ratio **(E, F)** of flue-cured tobacco plants under different treatments at Chuxiong and Qujing experimental sites, respectively. The different lowercase letters on the error bars show significant differences among treatments (As described in **Figure 1**) according to the least significant difference test (LSD; p< 0.05) after specific days of transplantation.

The application of biochar had a significant impact on the contents of soil organic carbon (SOC), total nitrogen (TN), and carbon/nitrogen (C/N) ratio of the flue-cured plants grown in paddy soil (Chuxiong) and red earth (Qujing) (**Figure 6**). At both experimental sites, after the application of biochar, contents of SOC first increased and then decreased with time (**Figures 6A, B**). The contents of SOC significantly increased with an increase in the application rate of biochar and were significantly higher in T4 (1800 kg/ha) than in control (CK; 0 kg/ha), T1 (600 kg/ha), T2 (900 kg/ha), and T3 (1200 kg/ha) (LSD; *p<* 0.05, **Figures 6A, B**). The content of TN was significantly higher in plants treated with biochar than in CK; however, there was no significant difference among the treatments (T1-T4). The contents of TN first increased and then decreased with time (**Figures 6C, D**). However, no significant difference was observed in the contents of TN at the Chuxiong experimental site after 35 days of transplantation under different treatments (CK, T1-T4; **Figures 6C, D**). The C/N ratio of flue-cured tobacco plants increased with the biochar application rate and was significantly higher in T4 than in CK, T1, T2, and T3 (**Figures 6E, F**).

### 3.8 Effect of different biochar dosages on the contents of nitrate nitrogen and ammonium nitrogen in the soil

**Figure 7:**
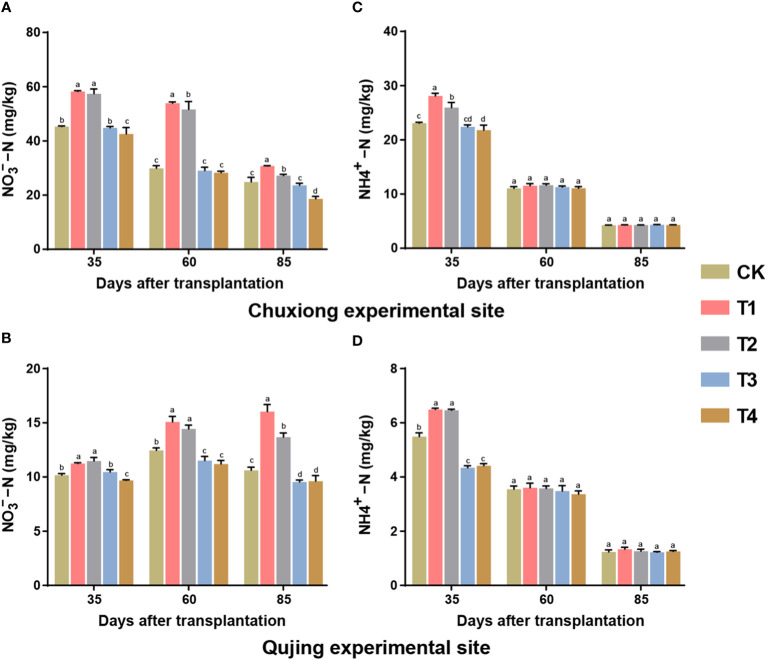
Effect of different application rates of biochar on contents of soil nitrate nitrogen (NO_3_^−^−N) and ammonium nitrogen (NH_4_^+^−N) of flue-cured tobacco plants. Bar plots top and bottom represent the contents of NO_3_^−^−N **(A, B)** and NH_4_^+^−N **(C, D)** of flue-cured tobacco plants under different treatments (As described in **Figure 1**) at the Chuxiong and Qujing experimental sites, respectively. The lowercase letters on the error bars represent the significant differences among treatments (As described in **Figure 1**) after specific days of transplantation according to the least significant difference test (LSD; *p*< 0.05).

The contents of nitrate nitrogen (NO_3_^−^−N) and ammonium nitrogen (NH_4_^+^−N) were significantly affected under different treatments of biochar (**Figure 7**). At both experimental sites, the contents of NO_3_^−^−N were significantly higher in treatment T1 (600 kg/ha) and T2 (900 kg/ha) compared with CK (0 kg/ha), T3 (1200 kg/ha), and T4 (1800 kg/ha) (LSD; *p<* 0.05, **Figures 7A, B**). While at the Chuxiong experimental site, the contents of NO_3_^−^−N first increased and then decreased with time. Meanwhile, at the Qujing experimental site, the contents of NO_3_^−^−N were increased over time. The contents of NH_4_^+^−N were found to be significantly higher in T1 and T2 than CK and T3-T4 at both experiment sites at 35 days of post-transplantation (**Figures 7C, D**). However, the contents of NH_4_^+^−N decreased in the soil after 60 and 85 days of transplantation at both experimental sites with the time, and no significant difference was observed among treatments. This suggested that the application of biochar at an optimum dosage increased the contents of NO_3_^−^−N and decreased under high dosage. In contrast, biochar application had no significant effect on the soil nutrient contents of NH_4_^+^−N during the growth period of flue-cured tobacco plants.

### 3.9 Correlation analysis between different indexes

**Figure 8:**
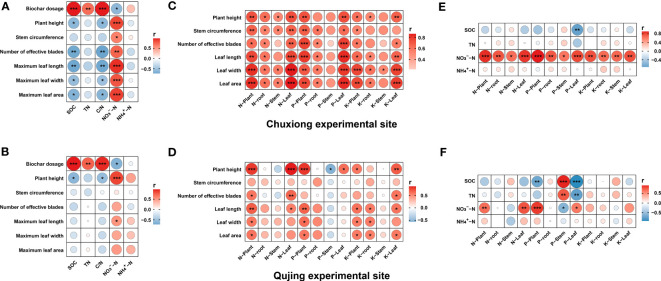
Relationship between biochar dosage, plant agronomic traits, N, P, K accumulation in different plant parts, and soil physicochemical properties. A significant Spearman’s correlation coefficients were noted by asterisks *p< 0.05, **p< 0.01, and ***p< 0.001. **(A, B)**; correlation analysis between biochar dosage, plant agronomic traits, and soil physiochemical properties, **(C, D)**; correlation analysis between plant agronomic traits and nutrient accumulation in different plant parts, and **(E, F)**; correlation analysis between soil physiochemical properties and nutrients accumulation in different plant parts.

Correlation analysis was conducted for biochar dosage, plant physiological traits, soil physicochemical properties, and nutrient accumulation in different plant parts to highlight the relationship among these indexes at both experimental sites (**Figure 8**). Results demonstrated that at both experimental sites, biochar dosage was significantly positively correlated with SOC, soil TN, and soil C/N and negatively correlated with soil NO_3_^−^-N, whereas no correlation was observed with NH_4_^+^-N. A significant negative correlation was observed between plant physiological traits and soil C/N, except for stem circumference at the Chuxiong experimental side. At the Qujing experimental site, a weak negative correlation was found between plant physiological traits and soil C/N, except for plant height, which showed a significant negative correlation with soil C/N ratio (**Figures 8A, B**). At the Chuxiong experimental site, a strong positive correlation was recorded between plant physiological traits and nutrient accumulation in different parts (plant, root, stem, and leaf) of tobacco plants, except for P and K in the stem (**Figure 8C**). However, in the Qujing experimental site, the correlation between these indexes varied to a certain degree, such as N, P, and K in the whole plant and leaf, which were positively correlated with plant height. In contrast, P in the stem is negatively correlated with plant height. No correlation was observed between stem circumference and nutrient accumulation in plant parts and for N, P, and K in root and stem with plant physiological traits except for P in stem with plant height (**Figure 8D**). Further correlation analysis between soil physicochemical properties and nutrient accumulation in plant parts revealed that in the Chuxiong experimental site, soil NO_3_^−^-N showed a significant positive correlation with nutrient accumulation in plant parts. However, no correlation was observed between soil TN, SOC, and NH4^+^-N and nutrient accumulation in all plant parts except for SOC, which showed a significant negative correlation with P in the leaf (**Figure 8E**). The relationship between soil physicochemical properties and nutrient accumulation in plant parts varied in the Qujing experimental site, where soil NO_3_^−^-N significantly positively correlated with N and P in plants and leaves and negatively correlated with P in stems. SOC showed a significant positive correlation with P in the stem and a negative correlation with P in the plant and leaf. Soil TN showed positive and negative correlation with P in stem and leaf, respectively, while no correlation was observed between soil NH4^+^-N and nutrient accumulation in plant parts (**Figure 8F**).

## 4 Discussion

Flue-cured tobacco is the main cash crop in Yunnan Province, China, which is famous for its unique taste, and its yield is affected by many abiotic and biotic factors (Ahmed et al., 2022a). Biochar is a carbon-rich source produced by the thermal decomposition of agricultural and industrial waste at high temperatures (Li et al., 2022). Soil amendment with biochar induces plant resistance against abiotic and biotic stresses, improves soil health and nutrient absorption ability, enhances plant growth and activity of microorganisms, and supports agricultural ecosystems (Ren et al., 2021; Li et al., 2022). In previous studies, biochar was mainly spread on the soil and then incorporated into the soil at a depth of ~20 cm (Hu et al., 2021). A large quantity of biochar was applied to be effective for the crops, which resulted in high input costs for the farmers. To reduce the input cost of biochar, in this study, biochar was directly applied in the holes (at the place of tobacco seedling transplantation) on ridges at different concentrations and evaluated its impact on soil nutrient contents, plant growth, and yield of flue-cured tobacco plants in two field experiments.

### 4.1 Effect of biochar dosage on the growth of flue-cured tobacco plants

The agronomic traits are important indicators for the growth, yield, and development of crop plants in the field (Khan et al., 2022; Wang et al., 2023). This study showed that after 35 days of transplantation at Chuxiong, the application of biochar inhibited the growth of the flue-cured tobacco plants, and the agronomic traits of flue-cured tobacco plants not treated with biochar (CK) were significantly higher than those treated with biochar. This may be due to the large specific surface area and cation exchange capacity of biochar, which can absorb organic matter and fertilizer in the soil to a certain extent and delay the release of fertilizer nutrients, thus inhibiting the growth of flue-cured tobacco at early growth stage (Dao et al., 2013). Meanwhile, at Qujing, no significant difference was observed for agronomic traits under all treatments (CK, T1-T4), which does not agree with the results from Chuxiong. The difference in growth response towards biochar application at both sites might be due to differences in soil properties and climatic variations. However, at 60 days of post-transplantation, tobacco plants at both test sites showed robust growth under low application rates (T1 and T2) of biochar, where the plant height, effective leaf number, and maximum leaf area were significantly higher than the plants treated with CK. These results indicate that biochar application within an appropriate range can substantially promote the growth of flue-cured tobacco in the middle and late-growth periods. Our results are in agreement with the previous studies that growth inhibition in the early growth periods of flue-cured tobacco plants after biochar application was fully recovered in the middle and late-growth periods (Pan et al., 2015; Jiang et al., 2018). In addition, we observed that under high biochar dosages (T3 and T4), the growth of tobacco plants was slower in the early, middle, and late stages, characterized by fewer leaves and smaller leaf areas. This might be due to the significant increase in soil carbon storage under high biochar application, resulting in the imbalance of soil C/N ratio, competition between microorganisms and plants for N, reduction of soil available N supply, N deficiency in tobacco plants, and ultimately inhibition of plant growth (Ali et al., 2020; Amoah-Antwi et al., 2020).

### 4.2 Impact of biochar dosage on SPAD values and nutrient uptake (N, P, and K) of tobacco plants

Chlorophyll is an essential pigment for photosynthesis in plants, and chlorophyll contents directly affect the photosynthetic capacity and accumulation of organic matter in leaves (Liu and Shi, 2010). It is reported that SPAD value (chlorophyll relative concentration) was positively correlated with chlorophyll contents and dry matter accumulation (Islam et al., 2014). In this study, we found that a low biochar dosage (T1 and T2) increased the SPAD value and the accumulation and distribution of N and K and improved the photosynthetic efficiency of tobacco leaves. This can be referred to as the biochar-mediated soil environment modulation and further enhancement in the absorption of nutrients by tobacco plants, thus improving photosynthetic efficiency (He et al., 2020). Generally, biochar application at low dosages (T1, T2) at both experimental sites were conducive to the N accumulation in the whole plant, stem, and leaf. In contrast, high dosages (T3 and T4) did not enhance the N accumulation except in the stem of tobacco plants grown at Qujing. A similar trend was recorded for the K accumulation under varying biochar dosages at both experimental sites. However, P accumulation was higher in whole plants and roots treated with T1 than in other treatments. The difference in nutrient accumulation can be attributed to the site effect, where soil and environmental conditions were not identical. It is reported that plant nutrient accumulation significantly affects the plant chlorophyll contents (Mauromicale et al., 2006). This supports our findings that an increase in the accumulation of nutrient contents in the leaves increases chlorophyll contents. In addition, we observed that the application of a high amount of biochar (T3 and T4) decreased the SPAD value and the N, P, and K contents in tobacco leaves. Previous studies have also reported that excessive biochar application is unfavorable for improving plants photosynthetic efficiency and may limit nutrient accumulation. The high C/N ratio may also lead to N fixation, thus affecting leaf photosynthesis (Asai et al., 2009; Liu et al., 2021). Therefore, it is concluded that excessive biochar application can reduce tobacco plant’s nutrient absorption and leaf photosynthesis.

### 4.3 Effect of biochar dosage on the biomass accumulation of flue-cured tobacco

The crop yield in the field depends upon the number of plants in the population and the growth of the individual. If the population size is the same, the yield of the field depends on the growth of the individual plants, and it is directly proportional to the accumulation of photosynthetic products (Dai et al., 2015). This study demonstrates that the application of biochar at different concentrations significantly affects the flue-cured tobacco plant biomass. Plant photosynthates are determined by many factors, including leaf area, light intensity, light duration, and chlorophyll contents (Ierna and Mauromicale, 2006). Our results showed that biochar could regulate the production of the photosynthetic products of flue-cured tobacco by affecting leaf area and chlorophyll content. In this study, under low biochar dosage (T1 and T2), the biomass accumulation increased significantly during the middle and late growth period of flue-cured tobacco plants, even though it was inhibited in the early growth stage, which is similar to the findings of Major et al. (2010). However, under a high application rate of biochar (T3 and T4), the growth of the flue-cured tobacco plant was inhibited during all the growth stages, and biomass accumulation also decreased significantly. This may be related to the nutrient loss caused by excessive biochar, which reduces the accumulation of nutrients in plants and is not conducive to efficient photosynthesis in plants, leading to a reduction in photosynthetic products (Wang et al., 2021). It may also be due to the significant increase in soil carbon storage under high biochar application, resulting in the imbalance of soil C/N ratio. Further, N competition between microorganisms and plants and the reduced available soil supply resulted in N deficiency of tobacco plants, inhibition of growth, and the subsequent reduction of biomass accumulation (Ali et al., 2020; Amoah-Antwi et al., 2020).

### 4.4 Effect of biochar dosage on the contents of SOC, TN, and its C/N ratio

Soil organic carbon contents and their balance are important indicators of soil quality or health, which directly controls soil fertility (Doran et al., 2018). In this study, we used biochar as a carbon source (57.83%) at different concentrations and found that biochar significantly impacted SOC contents. The contents of SOC proportionally increased at different growth periods of flue-cured tobacco plants, with an increase in biochar application rate. Our results correspond with the previous reports that biochar improves the SOC pool and inhibits the decomposition and transformation of soil organic matter (Cheng et al., 2008; Liu et al., 2016). A significant increase in the SOC pool might be due to the direct application of biochar in the holes (at the place of tobacco seedling transplantation) on the ridges, and soil samples were collected from the same location within a 15 cm diameter around the stem. Therefore, it is possible that the contents of SOC were recorded significantly higher under different treatments of biochar (T1-T4) compared with the control (**Figure 6**). However, further studies will focus on whether applying biochar in the holes on ridges can increase the SOC pool.

The contents of soil TN (sum of various forms of nitrogen) increased with an increase in the application rate of biochar, which is in accordance with the results of Liang et al. (2014) and Khan et al. (2021). Similarly, it is also reported that the contents of TN increased with the application of biochar because biochar itself contains a certain amount of readily and not readily decomposable N, and these two types of N were measured together for the determination of soil TN (Ouyang et al., 2014). In addition, we found that the soil C/N ratio significantly increased at different stages of tobacco growth with the application of higher concentrations of biochar. This may be because the adsorption of N by biochar is less than the direct supplement provided to SOC, and the increase of TN is less than that of SOC, significantly increasing the soil C/N ratio (Xu et al., 2021). Furthermore, the soil type also contributed to the variation of SOC and TN at both experimental sites. At Chuxiong, paddy soil had higher SOC and TN contents than red earth at the Qujing experimental site; therefore, the higher SOC and TN contents at Chuxiong might be attributed to already present SOC and TN contents in the soil. Previous studies have also reported that biochar application increased the SOC content in paddy soil (Xie et al., 2013; Jing et al., 2020). However, the biochar dosage positively correlated with SOC and TN at both experimental sites despite the different nutrient profiles of both soils (**Figures 8A, D**).

### 4.5 Effect of biochar dosage on soil contents of NO_3_^−^−N and NH_4_^+^−N

Plants can directly absorb and use soil mineral N, reflecting the short-term N availability in the soil. The results showed that the contents of NO_3_^−^−N in the soil increased at first and then decreased with the application of higher concentrations of biochar during the different growth periods of flue-cured tobacco plants. In this study, we found that the contents of NO_3_^−^−N were significantly increased at low concentrations (T1 and T2) of biochar (**Figure 7**); this might be due to the application of biochar at a certain level could enhance the N adsorption and retention, minimize N loss in the soil, promote ammonia oxidation to nitrite, and ultimately increase soil NO_3_^−^−N contents (Nelissen et al., 2012). However, the contents of NO_3_^−^−N significantly decreased under high dosages (T3 and T4) of biochar than CK; NO_3_^−^−N also showed a negative correlation with biochar dosage (**Figure 8**). It is known that due to excessive application of biochar, it tends to accumulate in the rhizosphere of plants, which inhibits soil microbial activity and reduces soil N mineralization, thus decreasing the soil NO_3_^−^−N contents (Luo et al., 2020). In addition, we found that biochar application had a negligible effect on soil NH_4_^+^−N contents, and no significant difference and correlation were observed for soil NH_4_^+^−N contents during different growth stages of flue-cured tobacco plants. This may be because biochar adsorbs harmful substances such as terpenes and phenol in the soil to promote soil nitrification, and NH_4_^+^−N in the soil can be transformed into organic N, NO_3_^−^−N, and other forms of N (Watson et al., 2017). Overall, the correlation analysis suggests that biochar dosage significantly influences the SOC, soil physicochemical properties, soil C/N ratio, and growth of tobacco plants. The SOC pool increased with the increased biochar application rate, which resulted in the imbalance of soil C/N ratio and inhibited tobacco plant growth.

## 5 Conclusions

In conclusion, high biochar application negatively affects soil nutrient contents, accumulation of nutrients in different plant parts, dry matter accumulation, and growth of flue-cured tobacco plants. Correlation analysis showed that SOC, soil TN, and soil C/N were significantly positively correlated with biochar dosages. Application of biochar at a certain level (600−900 kg/ha) could significantly increase the contents of SOC, TN, and NO_3_^−^−N, regulate the soil C/N ratio in an optimal range, promote the growth, increase the SPAD value of tobacco leaves, and improves the accumulation and distribution of N, P, K, and dry matter in tobacco leaves. However, under a high application rate of biochar (≥ 1200 kg/ha), the soil carbon pool was too high, which resulted in the imbalance of soil C/N ratio and reduced available N content and inhibited the nutrient supply and accumulation in the aboveground part of the plant. Consequently, plants showed a decrease in the number of leaves, photosynthetic leaf area, inefficient accumulation and distribution of nutrients (N, P, K), and decreased photosynthetic capacity of the plant, which ultimately led to reduced biomass and yield of the tobacco plants. Future studies must focus on unraveling the different biochar application rates and fertilization practices on the growth of tobacco plants at a molecular level and soil microbial diversity and on soil temperature, soil hydrology, and soil structure.

## Data availability statement

The raw data supporting the conclusions of this article will be made available by the authors, without undue reservation.

## Author contributions

ZZ conceived and designed the experiments. YY, CY, LY, LW, and XH performed the experiments. YY, ZD, WA, and XZ collected and analyzed the data. YY, WA, and KK wrote the manuscript. WA, KK, and ZZ revised the manuscript. All authors contributed to the final draft of the manuscript. All authors have read and agreed to the published version of the manuscript.
